# Postoperative Management of Anterior Visual Pathway Cavernoma, a Unique Perspective: Case Report

**DOI:** 10.7759/cureus.3819

**Published:** 2019-01-03

**Authors:** Brian DelPino, Jake Durden, Eric M Deshaies

**Affiliations:** 1 Neurological Surgery, Touro College of Osteopathic Medicine, New York, USA; 2 Neurological Surgery, Crouse Hospital, Syracuse, USA

**Keywords:** cavernous malformation, cavernous angioma, chiasmatic apoplexy, optic chiasm, optic nerve, anterior visual pathway

## Abstract

Cavernous malformations (CMs) are low-flow vascular lesions with an incidence of 0.1% to 0.7% in the general population. Less than 1% are found in the anterior visual pathway. The most common presenting symptoms are visual disturbances due to hemorrhage and the current standard of treatment is gross total resection.

The authors report a case of a 42-year-old male with visual disturbance and findings on T1-weighted magnetic resonance imaging (MRI) suggesting CM of the right optic nerve and right optic chiasm. The patient underwent right pterional craniotomy for gross total resection of the lesion. One year postoperatively, the patient demonstrated improvement in visual deficits with no signs of recurrence on MRI. Thirty-two months postresection, MRI showed a small slightly lobulated area of T1 hyperintense material within the postoperative cavity along the right aspect of the optic chiasm. MRI at 39 months postresection showed previously seen small amounts of T1 hyperintensity in the central and right aspect of the optic chiasm, with significantly decreased conspicuity. These findings suggest a trace amount of recurrence in the 32-month postoperative imaging despite overall stable visual field testing.

There is a paucity of literature concerning the retreatment of resected CM in the anterior visual pathway. The authors suggest serial imaging as an integral component of CM management. Although repeated visual field testing and clinical follow-up are important aspects of CM management, they are no substitute for the gold standard of MRI.

## Introduction

Cavernomas (cavernous malformations, CMs) are low-flow vascular lesions composed of a cluster of dilated capillaries with an endothelial lining and adventitia [1]. The majority of CMs are located supratentorially in the frontal and temporal areas [2-3]. Previous reviews have found CMs with an incidence of 0.3% to 0.7% in the general population [1,4-5], with <1% located in the optic pathway [1,6]. Patients most commonly present with visual disturbances due to bleeding. The standard treatment is gross total resection (GTR), which has enabled an improvement of symptoms in the majority of cases [1]. In this report, we present a rare case of CM of the right optic nerve and optic chiasm in order to review its clinical characteristics and supporting literature. The extensive duration of clinical follow-up and serial imaging in the present case provide a unique perspective on CM management.

## Case presentation

History and examination

**Figure 1:**
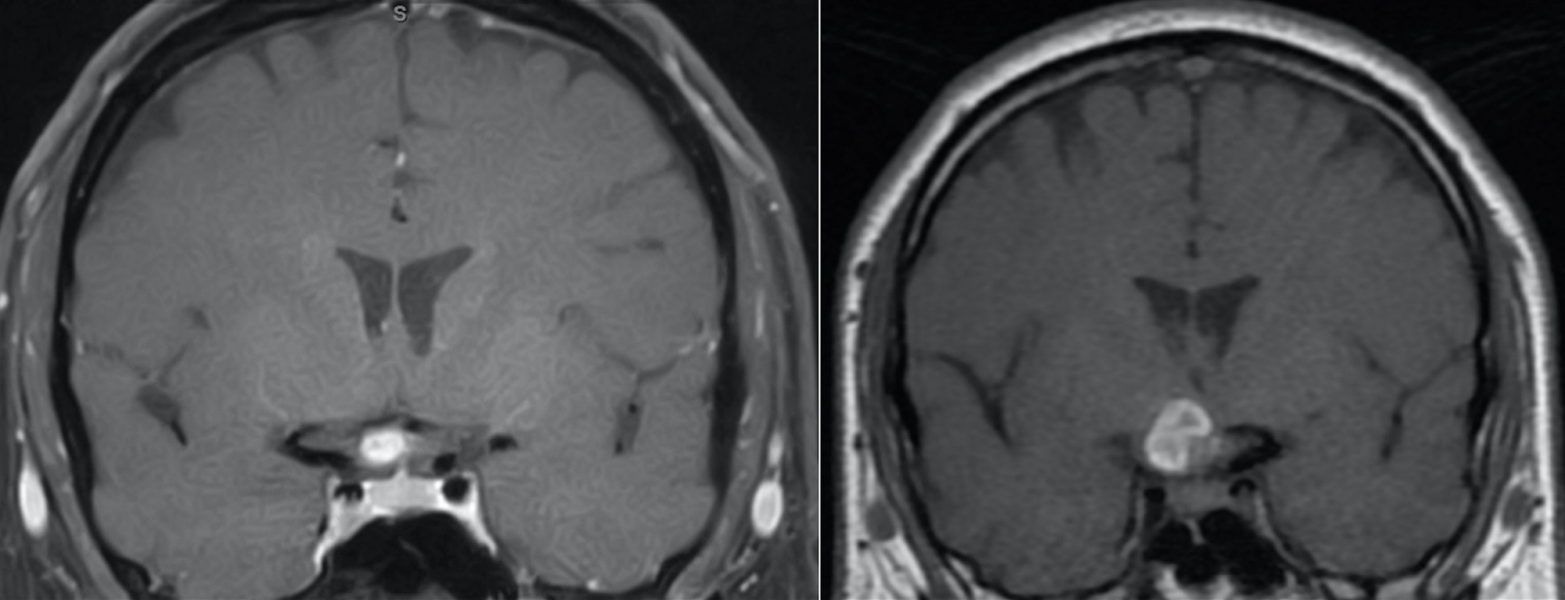
Coronal T1-weighted contrast MR image (left) showing hyperintensity along the right optic chiasm and (right) increase in size two months later, consistent with hemorrhagic CM MR: magnetic resonance; CM: cavernous malformation

**Figure 2:**
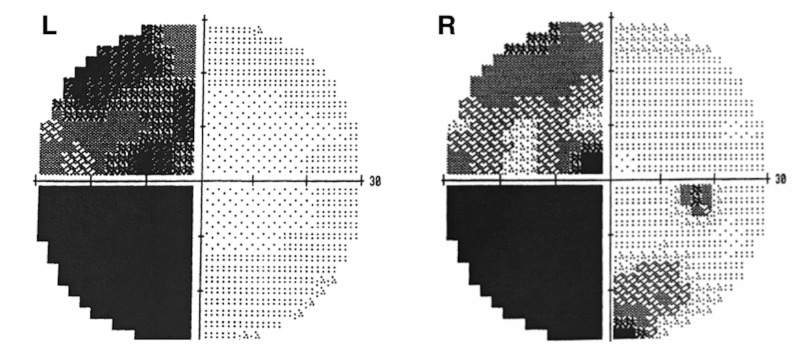
Visual field testing showing deficits in left temporal fields, right nasal fields, and right inferior temporal field

A 42-year-old male with a history of right eye visual field abnormalities presented with recent visual disturbances of the right eye and intermittent headaches. The visual disturbances were described as intermittent spots of blurriness. Initial MRI with contrast showed an oval-shaped lesion within or abutting the right optic chiasm. The lesion demonstrated intrinsic T1 hyperintensity (Figure 1), as well as susceptibility and increased T2 and fluid-attenuated inversion recovery (FLAIR) signal. In the subsequent months, the patient complained of worsening visual changes that included the left eye as well. Visual acuity was graded 20/30 OD, 20/20 OS, and visual field testing revealed a very small scotoma in the left lower quadrant of the right eye. Pupils were equal, round, and reactive to light. Color vision was within normal limits with 14 out of 14 color plates correctly named in each eye. A dilated funduscopic exam revealed the discs to be sharp and pink with a cup to disc ratio of 0.1 OD, 0.2 OS and no optic nerve pallor on either side. Extra-ocular motility was intact bilaterally. At this time, the patient was referred to our services and was diagnosed as a possible CM, with a differential diagnosis, including craniopharyngioma, meningioma, and arteriovenous malformation (AVM). Due to the eloquent location of the lesion and the risk of visual loss, observation was chosen over surgery. Two months after presentation to the clinic, the patient visited the emergency department due to headaches and further visual changes in the inferior fields of both eyes, citing increased blurriness specifically. Visual acuity worsened to 20/40 OD, with no visual field cuts and no papilledema bilaterally. The patient was started on corticosteroids. Repeat MRI showed an expansion of the right optic chiasm/nerve lesion with increased T1 hyperintensity compatible with acute hemorrhage (Figure 1). The lesion extended posteriorly and laterally to abut the right uncus and right cerebral peduncle. Visual field deficits were present in about three-quarters of his vision in both eyes, including the left temporal field and the right inferior nasal field (Figure 2). After discussing therapeutic strategies with the patient, the decision was made to operate because subsequent bleeding could have caused irreversible blindness in both eyes.

Operation

Under general anesthesia, the senior author (EMD) performed a right pterional craniotomy for the resection of the right optic nerve and chiasm CM. Brain relaxation was performed by draining cerebrospinal fluid from the cisterns, making the opening of the Sylvian fissure unnecessary. The right optic nerve was identified microscopically and followed back to the chiasm, which showed purplish discoloration with hemosiderin staining. Discoloration was distributed along the superomedial aspect of the optic nerve on the right side and the posterior aspect of the optic chiasm. The CM was microdissected from the surface of the optic nerve without incising the nerve itself. The middle portion of the CM was densely adherent to the vasculature of the optic chiasm and nerve. Cauterization of the residual portion of the CM was completed, preserving the vasculature in order to reduce the likelihood of an ischemic event. Frozen and permanent specimens were sent to pathology. The frozen section returned as abnormal vessel and hematoma. Surgically, there was GTR; however, subsequent radiographic imaging provided evidence that the resection may have been subtotal (see discussion). Craniotomy closure occurred by the replacement of the bone flap and the reapproximation of the myocutaneous flap.

Postoperative course

**Figure 3:**
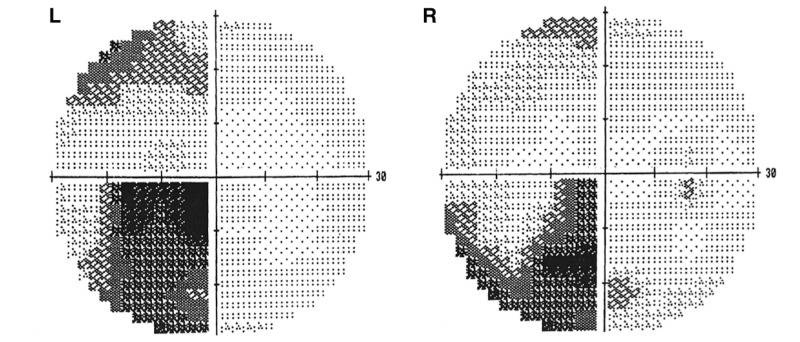
Visual field testing three weeks postoperatively showing improved vision in all visual fields

**Figure 4:**
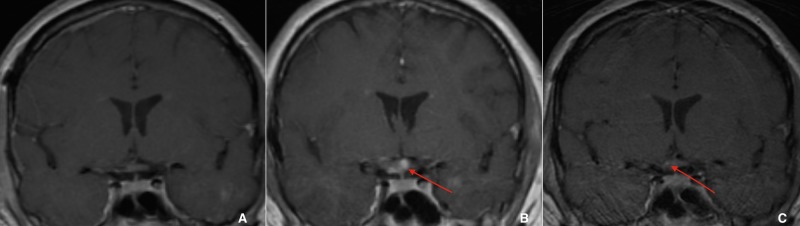
A: Coronal T1-weighted contrast MR image one year postoperatively shows complete resection of the right optic chiasm CM, seen by the absence of hyperintensity with no contrast on enhancement. B: Coronal T1-weighted MR image 32 months postoperatively shows the recurrence of hyperintensity in the right optic chiasm. C: Coronal T1-weighted MR image 39 months postoperatively shows a questionable area of small hyperintensity in the right optic chiasm MR: magnetic resonance; CM: cavernous malformation

There were no immediate complications following the procedure. The permanent specimen returned as CM and three weeks postoperatively, the patient had regained approximately half of the vision that was lost and continued to improve (Figure 3). MRI 12 months postoperatively showed no sign of recurrence (Figure 4A). Over two years after surgery, the patient had resumed all preoperative activities and reported significant visual recovery, with headaches occurring only once weekly. Thirty-two months after resection, MRI showed a small slightly lobulated area of T1 hyperintense material within the postoperative cavity along the right aspect of the optic chiasm (Figure 4B). This finding was new as compared to prior imaging and suggested that minimal recurrence in this location should be considered. Upon follow-up with ophthalmology, visual field deficits were stable. MRI at 39 months postresection showed previously seen small amounts of T1 hyperintensity in the central and right aspect of the optic chiasm with significantly decreased conspicuity (Figure 4C). Only a trace amount of T1 hyperintensity remained at the right aspect of the optic chiasm, suggesting the 32-month postop scan may have demonstrated a trace amount of subacute hemorrhage in the area suspected of being residual CM. During ophthalmological follow-up at three years, the patient mentioned having difficulty reading and more consistently occurring headaches since his office visit six months prior. Headaches were reported to be different than past migraines. When compared to older visual field testing, there was a worsening of deficits in the left eye but within the standard deviation. Visual acuity remained stable at 20/30 OD, 20/20 OS. The suspected residual CM will be followed with serial imaging and visual field tests with the possibility of additional surgical resection in the case of visual deterioration.

## Discussion

History and examination

In this report, we discuss a rare finding of CM of the optic chiasm and optic nerve. Previous case reports have found the incidence of the optic pathway CM to be the highest in the optic chiasm, followed by the optic nerve and optic tract [1]. The present case presented similarly to those found in the literature, with visual disturbances and headaches occurring due to recurrent hemorrhages and growth. This can be precipitated by the consumption of alcohol, pregnancy, and physical effort. Other common complications that have been reported are sudden frontal/retroorbital headaches, dizziness, subarachnoid hemorrhage, and hypothalamic-hypophyseal disorders [2,4,7]. There have also been reports of CM resulting in abducens nerve palsy [5,8].

Imaging

CM in the optic pathway is a diagnosis of exclusion that cannot be confirmed preoperatively. MRI is currently the best imaging modality for the identification of CM, with the highest sensitivity and specificity. In at least 55% of cases in a previous review, anterior visual pathway CM was not diagnosed on MRI due to diagnostic uncertainty [5]. Minimal to no enhancement occurs after the intravenous administration of gadolinium [1,3,9]. On the T1-weighted image, lesions appear hypointense while on T2, the lesions appear heterogenous with characteristic "popcorn" lesions demonstrating mixed hyperintense and hypointense signals [1]. On gradient-echo T2 imaging, hypointensity can be portrayed better due to hemosiderin deposition [3]. Gradient-echo sequences are particularly sensitive to small hemorrhages and have been demonstrated to be very useful for detecting the presence of additional lesions in patients with more than one CM [1,10]. CM of the optic nerve or tract can appear as a thickening of the nerve on coronal views whereas those in the chiasm can appear as focal and round masses that result due to decreased water motion within the lesion [1,11]. CM is one of the few optic pathway lesions that demonstrates a classical presentation on gradient-echo T2 imaging [11]. On computed tomography (CT), CMs appear as "well-demarcated hyperdense lesions with or without calcification." Angiography has been reported in previous literature as not very useful in diagnosis due to the characteristic low flow of the lesion and the high incidence of thrombosis [1-2,7-8].

Treatment

Previous reviews have found that surgery is the preferred treatment to prevent permanent visual damage in CM of the optic pathway, with GTR preferred, as subtotal resection (STR) may allow the regrowth of the CM [1,4-5,8]. Up to 94% of optic CMs treated surgically with GTR or STR reported visual improvement or stabilization [1]. When chiasmal apoplexy presents, surgical intervention is recommended to avoid permanent compressive damage to the visual pathway. The case described was thought to be managed by GTR. Although, with suggested findings of recurrence, it is possible that STR occurred.

Historically, surgical resection is difficult due to the deep location of the lesion as well as the firm adhesion of the capsule. A review of optic CM approaches discussed a wide range of opinions on the various approaches to surgical resection, with anterolateral approaches being the most prevalent. There have been many reports of successful surgical resection with the frontotemporal, pterional, such as our case, and orbitozygomatic approaches. The orbitozygomatic approach particularly offers more exposure and a shorter distance to the lesion [1].

Recently, Meng et al. used an "endoscopic endonasal transsphenoidal approach" (EETA), which may have advantages such as the avoidance of excessive dissection of structures, for example, the anterior communicating artery, optic nerves, and chiasm. The endoscopic technique is also beneficial because it offers a close-up panoramic view at different angles and avoids visual field limitations. Finally, vascular supply to the chiasm is supplied from bilateral internal carotid arteries and the anterior communicating artery complex [6,12]. EETA can be performed ventrally to avoid the lateral arteries while protecting the superior arterial supply directly, which might lead to better postoperative improvement in vision. More cases are needed to compare EETA with traditional surgical approaches.

There is a consensus that radiosurgery should be excluded as a treatment method to avoid subsequent bleeding and damage to the optic pathways [2,13-14].

## Conclusions

In the present case, follow-up imaging showed no significant abnormalities until 32 months after what appeared intraoperatively to be GTR. This raises the questions of possible recurrences years after apparent GTR and how best to manage lesions that have already once thwarted surgical resection. There is a paucity of literature concerning the retreatment of resected CM in the anterior visual pathway. The authors suggest serial imaging as an integral component of CM management. Although repeated visual field testing and clinical follow-up are important aspects of CM management, they are no substitute for the gold standard of MRI. The current case demonstrates the difficulty of drawing conclusions from visual field testing, as results can fluctuate considerably while remaining within the standard deviation, preventing definitive conclusions from being drawn. Serial imaging can corroborate clinical symptoms with changes in the appearance of the lesion and suspected hemorrhage.
